# Effect of 1-MHz ultrasound on the proinflammatory interleukin-6 secretion in human keratinocytes

**DOI:** 10.1038/s41598-021-98141-2

**Published:** 2021-09-24

**Authors:** Sabrina Giantulli, Elisabetta Tortorella, Francesco Brasili, Susanna Scarpa, Barbara Cerroni, Gaio Paradossi, Angelico Bedini, Stefania Morrone, Ida Silvestri, Fabio Domenici

**Affiliations:** 1 Department of Molecular Medicine, Sapienza University of Rome, Rome, Italy; 2 Department of Chemical Sciences and Technologies, University of Rome Tor Vergata, Rome, Italy; 3 Department of Physics, Sapienza University of Rome, Rome, Italy; 4 CNR-NANOTEC, Institute of Nanotechnology, Soft and Living Matter Laboratory, Rome, Italy; 5 Department of Experimental Medicine, Sapienza University of Rome, Rome, Italy; 6 INAIL, Italian Worker’s Compensation Authority, Rome, Italy

**Keywords:** Biophysics, Health care, Health occupations

## Abstract

Keratinocytes, the main cell type of the skin, are one of the most exposed cells to environmental factors, providing a first defence barrier for the host and actively participating in immune response. In fact, keratinocytes express pattern recognition receptors that interact with pathogen associated molecular patterns and damage associated molecular patterns, leading to the production of cytokines and chemokines, including interleukin (IL)-6. Herein, we investigated whether mechanical energy transported by low intensity ultrasound (US) could generate a mechanical stress able to induce the release of inflammatory cytokine such IL-6 in the human keratinocyte cell line, HaCaT. The extensive clinical application of US in both diagnosis and therapy suggests the need to better understand the related biological effects. Our results point out that US promotes the overexpression and secretion of IL-6, associated with the activation of nuclear factor-κB (NF-κB). Furthermore, we observed a reduced cell viability dependent on exposure parameters together with alterations in membrane permeability, paving the way for further investigating the molecular mechanisms related to US exposure.

## Introduction

There is a growing interest of the scientific community in the applications of ultrasound (US) in medicine for diagnostic and therapeutic purposes. In particular, unfocused low-intensity pulsed US (LIPUS) at the frequency of 1 MHz has been widely applied for many years for physical therapy, to provide either thermal effects on a painful body part or micromechanical inputs, the latter to favour the drug delivery into cells (i.e., sonoporation) and to overcome tight junctions among endothelial cells (i.e., sonophoresis)^1–5^.

The biological risk level of treatments by megasonic energy fields is believed to be low. Nonetheless, when the energy exceeds the cavitation threshold, the production of gas microbubbles negatively affects cells and tissues^6,7^. However, biological alterations have also been detected at low intensities below the regime of cavitation^8–10^, particularly at 1 MHz and spatial peak temporal average intensity (I_spta_) below 100 mW/cm^2^. According to the bilayer sonophore model^11^, the mechanical energy transported by a low intensity US field is absorbed by the cell membranes, inducing contraction and expansion of the intermembrane space. This entails a reversible, temporary enhancement of the plasma membrane permeability of the blood–brain barrier and skin^12,13^.

Regardless of its medical use, US necessarily involves the transfer of mechanical energy within skin cells, which may undergo transient micromechanical stress and wild type permeability alterations. These alterations are expected to trigger the activation of acute proinflammatory responses^14,15^, resembling what happens when tissue cells undergo mechanical insults such as shearing and tensile straining^16,17^. Our appealing hypothesis in this regard is that mechanical energy provided by low intensity US can produce transient, non-negligible cellular alterations on human skin.

The skin represents a barrier that provides the first line of defence against environmental factors^18^. A fine equilibrium among different cellular populations and microbial components allows to maintain and restore tissue homeostasis. Dysregulation of this equilibrium contributes to the pathogenesis of inflammatory skin diseases^19^, resulting in local and systemic clinical manifestations^20^.

The epithelium and the connective tissue are the two major components of the skin. Keratinocytes represent the most numerous cell types in the epithelial compartment. Melanocytes, immune cells^21^, such as Langerhans cells, other dendritic cells and T cells as well as Merkel cells are also present.

Keratinocytes not only provide a first line of defence barrier for the host but also actively participate in immune response^21^. It has been shown that keratinocytes can recognise pathogen associated molecular patterns (PAMPs) and damage associated molecular patterns (DAMPs) through pattern recognition receptors (PRRs), including toll-like receptors (TLRs), predominantly leading to the activation of Type 1T helper (Th1) cells^22^. Furthermore, PAMPs and DAMPs can activate a proinflammatory signalling pathway through the inflammasome after the interaction with nucleotide-binding oligomerisation domain-like receptors (NLR, NOD-like receptor)^23^. Receptor engagement leads to the activation of downstream signalling molecules, such as Nuclear Factor-kappa B (NF-κB)^24–26^. NF-κB promotes the expression of target genes including those for inflammatory cytokines such as interleukins (ILs) IL-1, IL-6, IL-8, IL-10, IL-18, IL-20, tumour necrosis factor (TNF-α) and chemokines^27,28^. Moreover, keratinocyte death is considered a potent trigger of skin inflammation^29^.

Inflammatory cytokines are up-regulated and released by keratinocytes under several different biological, chemical and physical stimuli^28^. In particular, IL-6 has a crucial role in various inflammatory skin diseases related to immune modulation, as well as in oncogenesis and differentiation^30,31^. In recent years, evidence has been collected that IL-6 production in human keratinocytes is stimulated by a wide range of inducers such as IL-1α, TLR ligands and ultraviolet (UV) radiation^21,32–35^.

Several studies have been conducted to evaluate the effects of mechanical stress on cell behaviour. The micromechanical energy transfer within cells can induce transient stress at the level of membrane permeability with consequent modifications of the integrin-actin organisation, linked to the effects of sonoporation and sonophoresis. These membrane alterations can trigger the activation of acute proinflammatory cytokines such as IL-6^36,37^. For this reason, we focused on the up-regulation and protein secretion of IL-6.

It is known that IL-6 is a pleiotropic cytokine that orchestrates a plethora of pro- and anti-inflammatory functions. In this respect, the aim of this study was to investigate whether low energy US could also represent a stimulus to induce a response in keratinocytes, the first cells involved during non-invasive medical treatments with 1 MHz US. It is known that damages induced by US exposure are dependent on their energy. In our previous study, we already detected that very low intensity US enhance membrane permeability and preliminary data suggested the possible activation of inflammatory pathways^38^.

In this study, we investigated firstly if the mechanical stress applied by the medically relevant 1 MHz LIPUS, being able to induce significant transient plasma membrane sonoporation, can also alter the expression of the inflammatory cytokine IL-6 in human keratinocytes. The effects were tested on HaCaT cells, a spontaneously immortalised cell line derived from normal human skin^39^. These cells have many similarities with primary basal keratinocytes, including the capability to produce many cytokines. It is reported that primary keratinocytes may show a change in the susceptibility to treatments with the increasing number of passages, whereas HaCaT cells ensure a higher reproducibility^40,41^. For these reasons HaCaT cells are largely used as experimental models.

In this frame, we felt it important to consider possible related activation of NF-κB and Signal Transducers and Activators of Transcription 3 (STAT3) proteins. Indeed, NF-κB coordinates the expression of different proinflammatory genes, IL-6, TNF-α, as well as the peroxisome proliferator-activated receptor (PPAR)-α. On the other hand, STAT3 transiently activated by different stimuli, including IL-6, can promote oncogenesis and auto-immune disease^41–44^, and has an important role in cell growth and apoptosis. In this connection, we also show the effects of US exposure on cell proliferation and the expression of the apoptosis-regulating genes, B cell lymphoma (BCL)-2 and BCL-2-associated X protein (BAX), in HaCaT cells at varying exposure parameters.

The discussion of the in vitro results concerning the activation of the IL-6 signalling pathway in HaCaT cells are supported by comparative evidence of transient morphological and permeability variations of plasma membranes, and by additional investigation on the effects of low intensity US on IL-1α protein expression level, which secretion is promoted by severe mechanical deformation^45–47^.

## Results

### Effect of US on IL-6 expression

**Figure 1:**
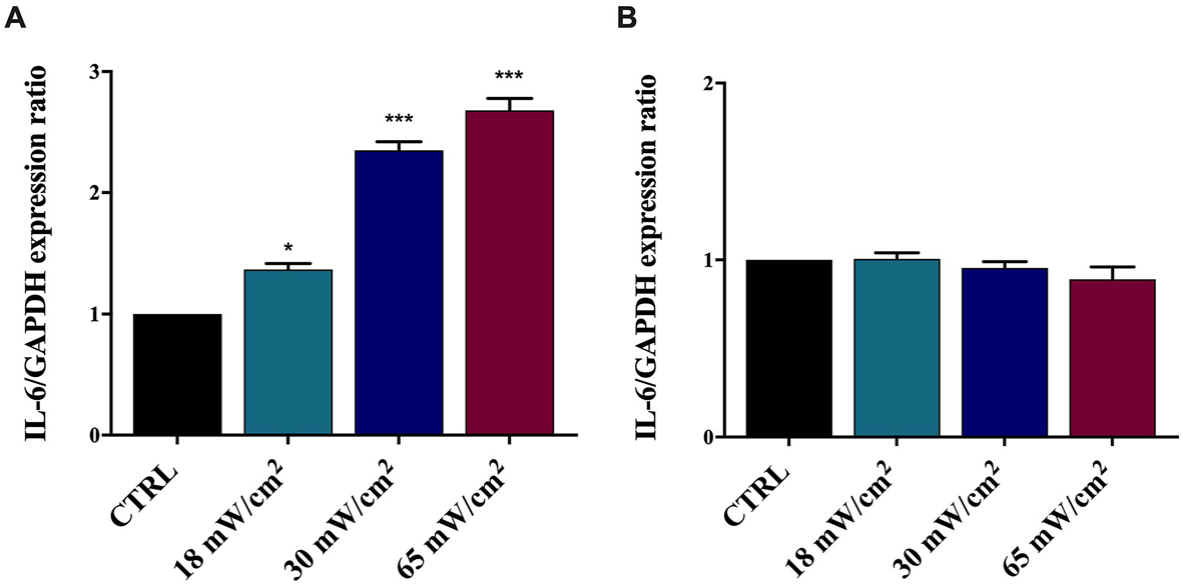
Real-time PCR analysis of IL-6 gene expression in HaCaT cells irradiated at 1 MHz for 1 h at varying I_spta_ values and analysed at 0 (**A**) and 24 h (**B**) from the treatment. IL-6 gene expression was normalised to GAPDH, used as reference gene. A significant increase of IL-6 mRNA level was observed in HaCaT cells immediately after irradiation (**A**) but not after 24 h (**B**). The reported values and error bars represent the average and the standard deviation evaluated on each sample analysed. Asterisks indicate significant difference from the control sample (*p < 0.05, ***p < 0.001). The graph is representative of at least three independent experiments.

HaCaT cells express and secrete IL-6^36^. After exposure to US for 1 h at 1 MHz (I_spta_ between 18 and 65 mW/cm^2^) these cells were examined for IL-6 expression at 0 and 24 h from the treatment by Real-time PCR. The obtained results are reported in Fig. 1. A significant increase in the IL-6 gene expression was detected at increasing I_spta_ values (panel A), with markedly up-regulated IL-6 mRNA at I_spta_ = 65 mW/cm^2^, as compared to the control sample, which was maintained at the same environmental conditions of treated samples for an amount of time corresponding to US exposure time. However, after 24 h we observed no statistically significant changes in the IL-6 mRNA expression (panel B), suggesting that low intensity 1 MHz US can induce a transient IL-6 up-regulation.

### Effect of US on IL-6 secretion

**Figure 2:**
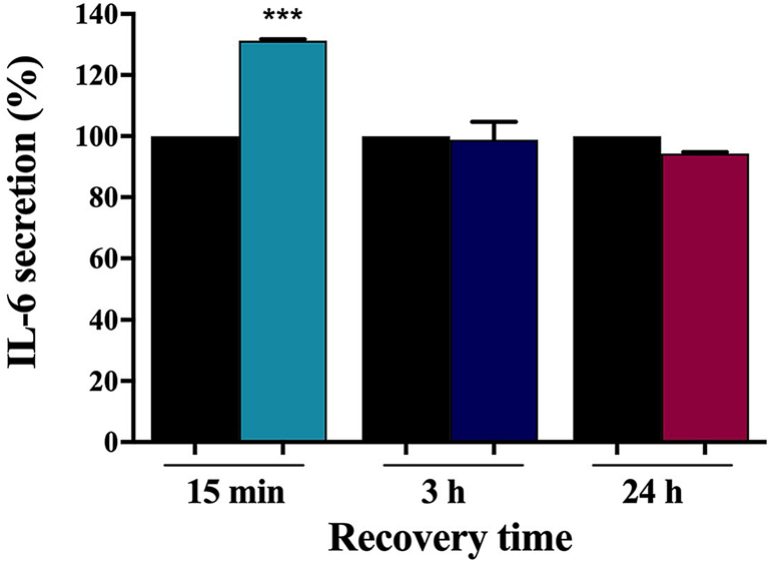
IL-6 secretion analysed by ELISA test in cultured media of HaCaT cells exposed for 1 h to 1 MHz US at I_spta_ = 65 mW/cm^2^ followed by different post-treatment recovery times. The control samples (black histograms) were maintained in the same environmental conditions of treated samples for an amount of time corresponding to exposure time. US stimulus drove a quick cytokine release at 15 min from the exposure. The reported values and error bars represent the average and the standard deviation evaluated on each sample analysed. Asterisks indicate significant difference from control samples (*** p < 0.001). The graph is representative of at least three independent experiments.

An ELISA test was performed after 1 h exposure to 1 MHz US at I_spta_ = 65 mW/cm^2^ and at different times of recovery^48^, to evaluate the effects of low intensity US on IL-6 secretion in HaCaT cells cultured media. As shown in Fig. 2, after 15 min of recovery the release of IL-6 from treated cells was 27% greater than untreated control cells. On the other hand, no statistically significant differences in IL-6 release were found after 3 and 24 h of recovery. These results suggest that 1 MHz US can induce a transient early IL-6 release, in accordance with the enhancement of IL-6 mRNA expression highlighted by real-time PCR.

It is well known that keratinocytes constitutively produce the cytokine IL-1α^45^, which can modulate the expression of genes involved in proliferation, differentiation, and cell adhesion. In addition, cytokine IL-1α is an essential cell-autonomous regulator and plays a fundamental role in the regulation of inflammatory processes. Several studies reported that a deregulated expression of IL-1α promotes inflammatory skin phenotype and is associated with numerous auto-inflammatory disorders. In this framework, a further ELISA test was performed to evaluate the ability of US stimulus to induce IL-1α secretion in HaCaT cells. No statistically significant differences were observed in the IL-1α levels (Figure S1 of Electronic Supplementary Information, ESI) in HaCaT cells irradiated at I_spta_ = 65 mW/cm^2^ for 1 h, with the exception of a slight decrease after 3 h of recovery.

### US exposure induces NF-κB activation

**Figure 3:**
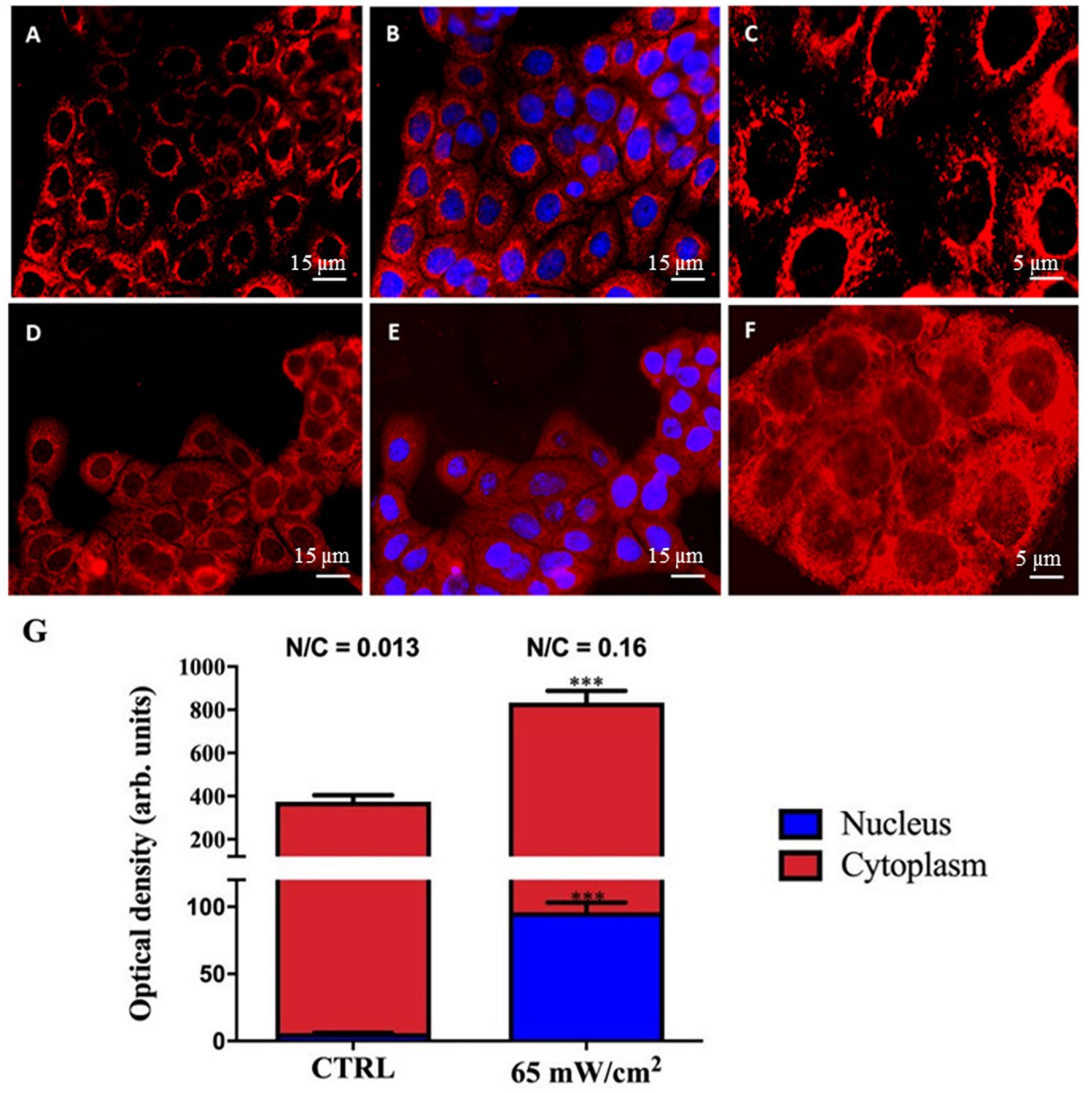
Immunofluorescence analysis of NF-κB activation in HaCaT cells exposed to 1 MHz US. HaCaT cells were stained with anti-p65 NF-κB (red) and Hoechst (blue). Representative images of the control sample (**A**–**C**) and of cells treated for 1 h at I_spta_ = 65 mW/cm^2^ (**D**–**F**) are reported. Scale bars measure 15 μm (**A**,**B**,**D**,**E**) and 5 μm (**C**,**F**). Densitometry measurements of the NF-κB immunofluorescent staining into the nucleus and into the cytoplasm are reported in panel (**G**). The ratio N/C between the two values is also reported above each column. The reported values and error bars represent the average and the standard deviation evaluated on ten different regions for each sample analysed. Asterisks indicate significant difference from control samples (***p < 0.001). The graph is representative of at least three independent experiments.

NF-κB accumulates in the cytoplasm at the steady state and translocates into the nucleus upon activation, where it can regulate the transcription of several genes such as IL-6^24^. Cytoplasmic and nuclear localisation of immunofluorescent NF-κB was analysed on HaCaT cells after exposure to 1 MHz US for 1 h at I_spta_ = 65 mW/cm^2^, where the deregulation of IL-6 production is revealed. The immunofluorescence staining results are shown in Fig. 3A–F. Untreated HaCaT cells showed an expression of NF-κB p65 in approximately 100% of the cells and the staining was found exclusively into the cytosol (Fig. 3A–C); after US exposure, the amount of cytoplasmic NF-κB p65 increased and it was possible to recognise NF-κB p65 also inside the nucleus of every cell (Fig. 3D–F). The densitometric quantification of immunofluorescent staining was performed and cytoplasmic NF-κB resulted 369 ± 30 densitometric units (DU) in untreated cells and 738 ± 54 DU in exposed cells, while nuclear NF-κB was 6.0 ± 0.3 in untreated cells and 96 ± 7 in exposed cells (Fig. 3G). The elaboration of these data showed a significant increase of nuclear/cytoplasmic ratio of NF-κB from 0.016 in the untreated control cells to 0.130 in the cells exposed to US. This last data demonstrated the translocation of NF-κB into the nucleus and its consequent activation after US exposure.

In addition, we evaluated the effects of 1 MHz US on the protein expression levels of STAT3. It is well known that STAT3 and NF-κB can cooperate to promote the target gene expression, and many cytokines, such as IL-6, can induce a feedback on STAT3 and NF-κB^49,50^. As reported in Figure S2 of ESI, we observed a decrease in the phosphorylated (p)-STAT3/STAT3 ratio after 1 h exposure at I_spta_ = 65 mW/cm^2^, followed by 15 min of recovery, with a slight increase after 3 h of recovery (further details are reported in Section S2 of ESI).

### Effects on viability and cell cycle

**Figure 4:**
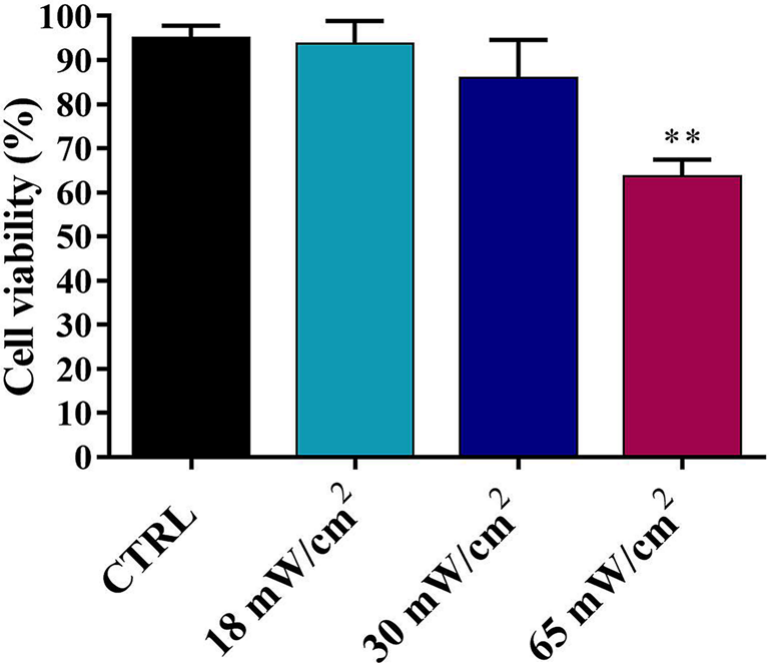
Cell proliferation analysis by MTT test on HaCaT cells exposed to 1 MHz US with I_spta_ 18, 30 and 65 mW/cm^2^ for 1 h. The reported values and error bars represent the average and the standard deviation evaluated on each sample analysed. Asterisks indicate significant difference from the control sample (**p < 0.01). The graph is representative of at least three independent experiments.

Cell proliferation was first evaluated by MTT (3-(4,5-dimethylthiazol-2-yl)-2,5-diphenyltetrazolium bromide) viability test as reported in Fig. 4, highlighting a significant decrease after the treatment at I_spta_ = 65 mW/cm^2^.

**Figure 5:**
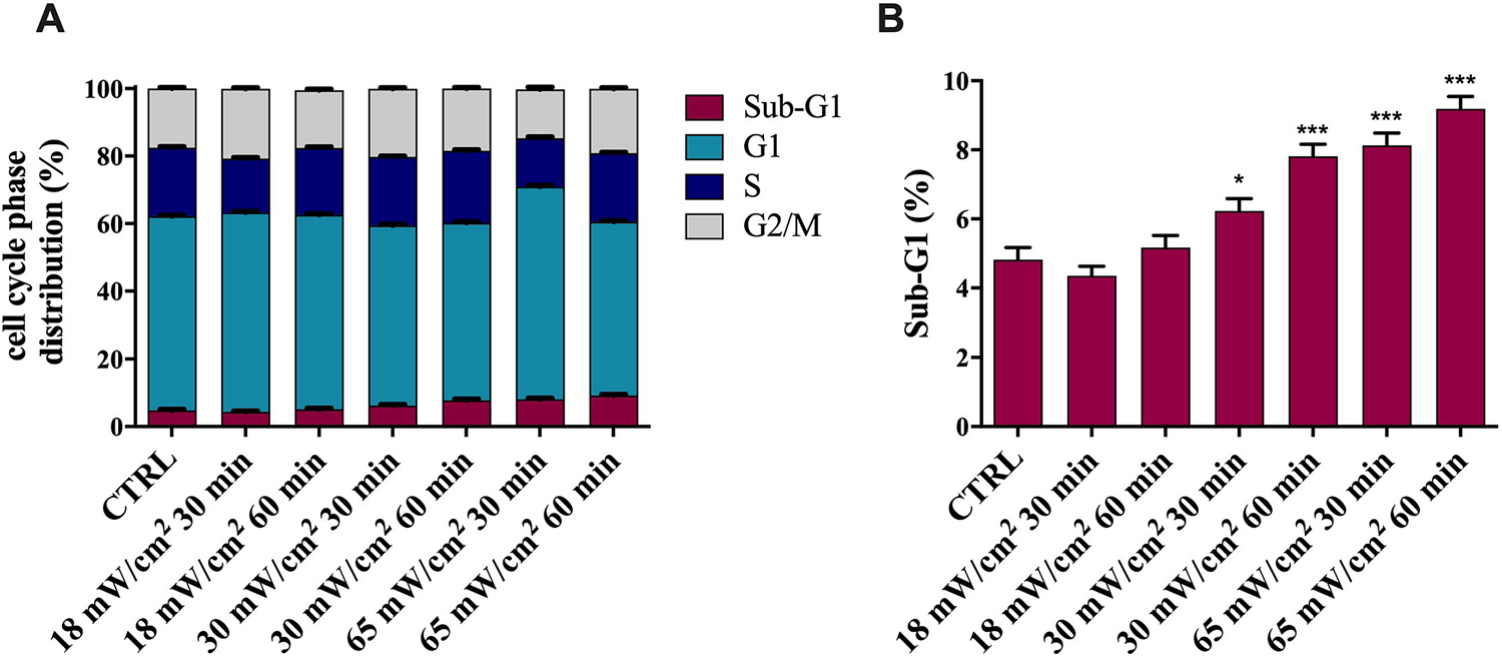
Cell cycle analysis on HaCaT cells exposed to at 1 MHz US for 30 and 60 min at different values of I_spta_ (**A**), together with the chart of the % Sub-G1 phase (**B**). No differences in cell cycle distribution were observed in the control samples analysed at 30 and 60 min. The reported values and error bars represent the average and the standard deviation evaluated on each sample analysed. Asterisks indicate significant difference from the control sample (*p < 0.05, ***p < 0.001). The graph is representative of at least three independent experiments.

The effects of 1 MHz US on cell cycle were evaluated at varying intensity and exposure time by flow cytometry analysis, as reported in Fig. 5. Treated cells showed significant differences in the distribution of cell cycle phases, depending on exposure parameters. Specifically, we observed a slight increase of the percentage of cells in Sub-G1 peak starting from I_spta_ = 30 mW/cm^2^ and 30 min of exposure (panel A,B).

To further analyse the effects of US on the cell viability and whether the increase of Sub-G1 peak detected in HaCaT cells after irradiation was associated with apoptosis, a flow cytometric analysis was performed using Annexin V/Propidium Iodide assay. As shown in Figure S3 of ESI, such a LIPUS stimulus induced a slight increase of the percentage of HaCaT cells undergoing late-stage apoptosis.

### Effects on BAX/BCL-2 mRNA expression

It is well known that the BCL-2 family proteins have an important role in the mitochondrial-mediated pathway of apoptosis. BAX and BCL-2 are considered the major members of this family, where BAX promotes apoptotic processes, whereas BCL-2 shows an antiapoptotic activity. The balance between BAX and BCL-2 factors regulates the apoptotic pathways.

**Figure 6:**
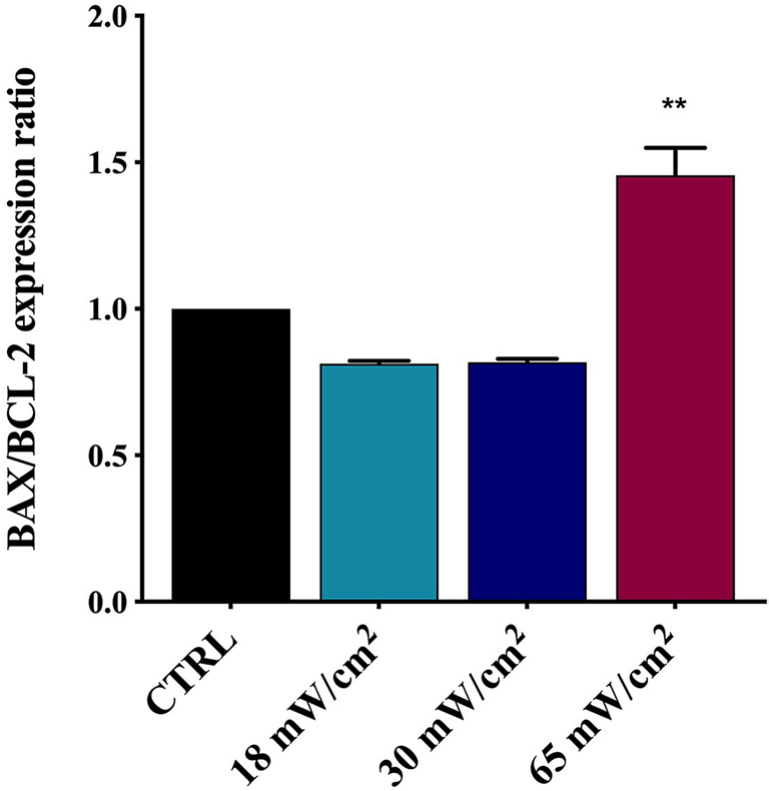
Effect of US treatment on BAX/BCL-2 gene expression ratio in HaCaT cells determined by real-time PCR and normalised to GAPDH as reference gene. HaCaT cells were treated for 60 min with 1 MHz at I_spta_ values between 18 and 65 mW/cm^2^. The analysis shows a statistically significant increase of BAX/BCL-2 ratio suggesting cell apoptosis after the treatment with Ispta = 65 mW/cm^2^. The reported values and error bars represent the average and the standard deviation evaluated on each sample analysed. Asterisks indicate significant difference from the control sample (**p < 0.01). The graph is representative of at least three independent experiments.

The BAX and BCL-2 gene expression (Fig. 6) was analysed by Real-time PCR. At higher I_spta_ values, the BAX mRNA resulted up-regulated after US exposure, with an increase of BAX/BCL-2 ratio, suggesting that a small number of cells underwent apoptosis.

### Study of the membrane permeability and morphology

We studied by confocal laser scanning microscopy (CLSM) US-induced alterations in the plasma membrane permeability and morphology of HaCaT cells, with the appealing hypothesis that the two phenomena might be related^51^ and might even lead to a transient proinflammatory interleukin response^36^.

**Figure 7:**
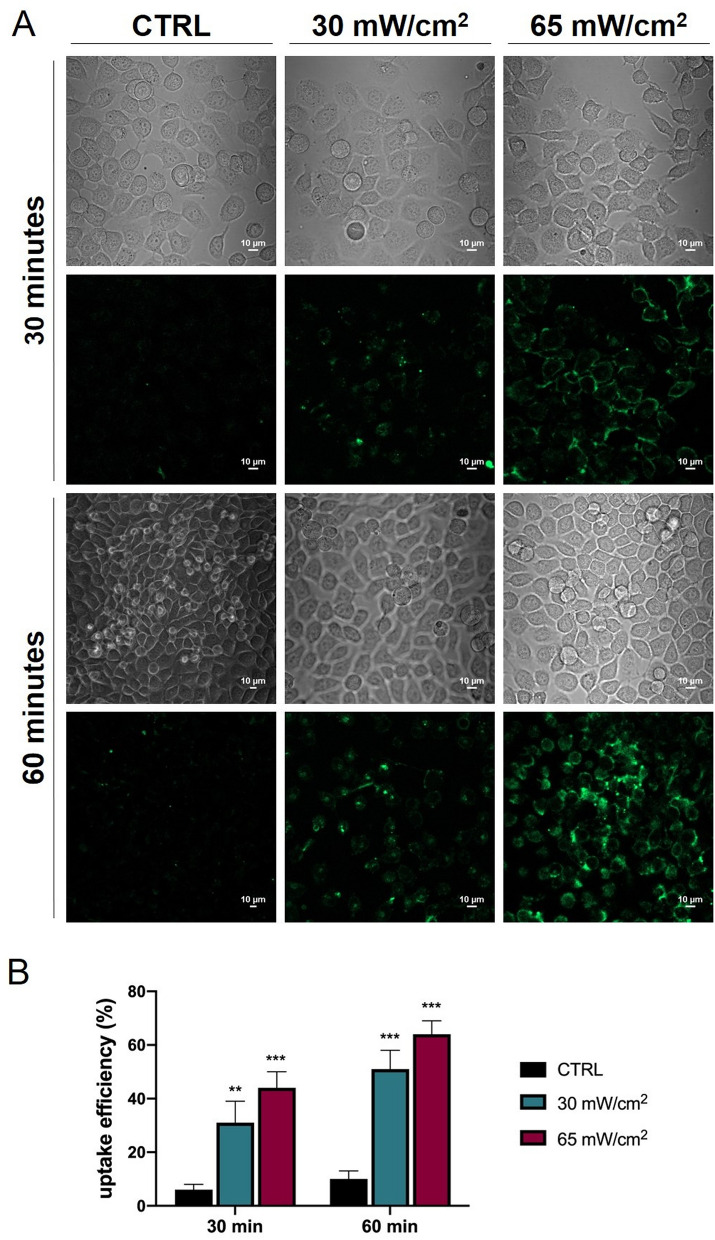
CLSM analysis of the calcein internalisation within HaCaT cells. (**A**) Representative images acquired on samples treated with 1 MHz US with different spatial peak temporal average intensities and exposure times. Each image is representative of the acquisition of different areas of the HaCaT cell culture and represents the results of a triplicate experiment. (**B**) Histogram of the uptake efficiency evaluated for the different exposure conditions. The reported values and error bars represent the average and the standard deviation of the uptake efficiency evaluated on the different regions and samples analysed. Asterisks indicate significant difference from the control sample (**p < 0.01, ***p < 0.001).

The cell membrane permeability was analysed by evaluating the cell internalisation of calcein, a green fluoroprobe for the membrane integrity^8^. We selected the same exposure conditions (I_spta_ and treatment time) that induced either a deregulation of the cell cycle progression or an up-regulation of IL-6 expression. Representative CLSM and transmission microscopy images of each sample are reported in Fig. 7, together with the histogram of the uptake efficiency evaluated as the percentage of the cells in the sample in which it is possible to individuate internalised calcein. Experiments highlight that the calcein uptake efficiency increases both with I_spta_ and treatment time. Specifically, in cells treated for 30 and 60 min, we evaluated the uptake efficiency of 31 ± 8% and 51 ± 7% at 30 mW/cm^2^ and of 44 ± 6% and 64 ± 5% at 65 mW/cm^2^, respectively. The internalised dye appears mainly localised in the plasma membrane and peripheral cytosol regions. Nevertheless, in samples treated with the highest dose (I_spta_ = 65 mW/cm^2^, 60 min), a second level of internalisation can be recognised, with calcein distributed deeper into the cytoplasm.

Alongside the membrane permeability, we studied possible transient changes in the shape or position of the cell membrane immediately after US exposure. Morphological changes can be monitored in real time (without cell fixation) by labelling the cell membrane with the red dye 1,1′-dioctadecyl-3,3,3′,3′-tetramethylindocarbocyanine perchlorate (DiI, Sigma-Aldrich Italy, excitation and emission wavelengths at 549 and 565 nm, respectively) and acquiring a time-progression of CLSM sections of cells immediately after exposure to US, while maintaining the focus rigorously fixed in the same position. This is accomplished thanks to the z-focus driving motor of our microscope, endowed with a linear encoder and an anti-vibration support, that ensures 500 nm resolution.

**Figure 8:**
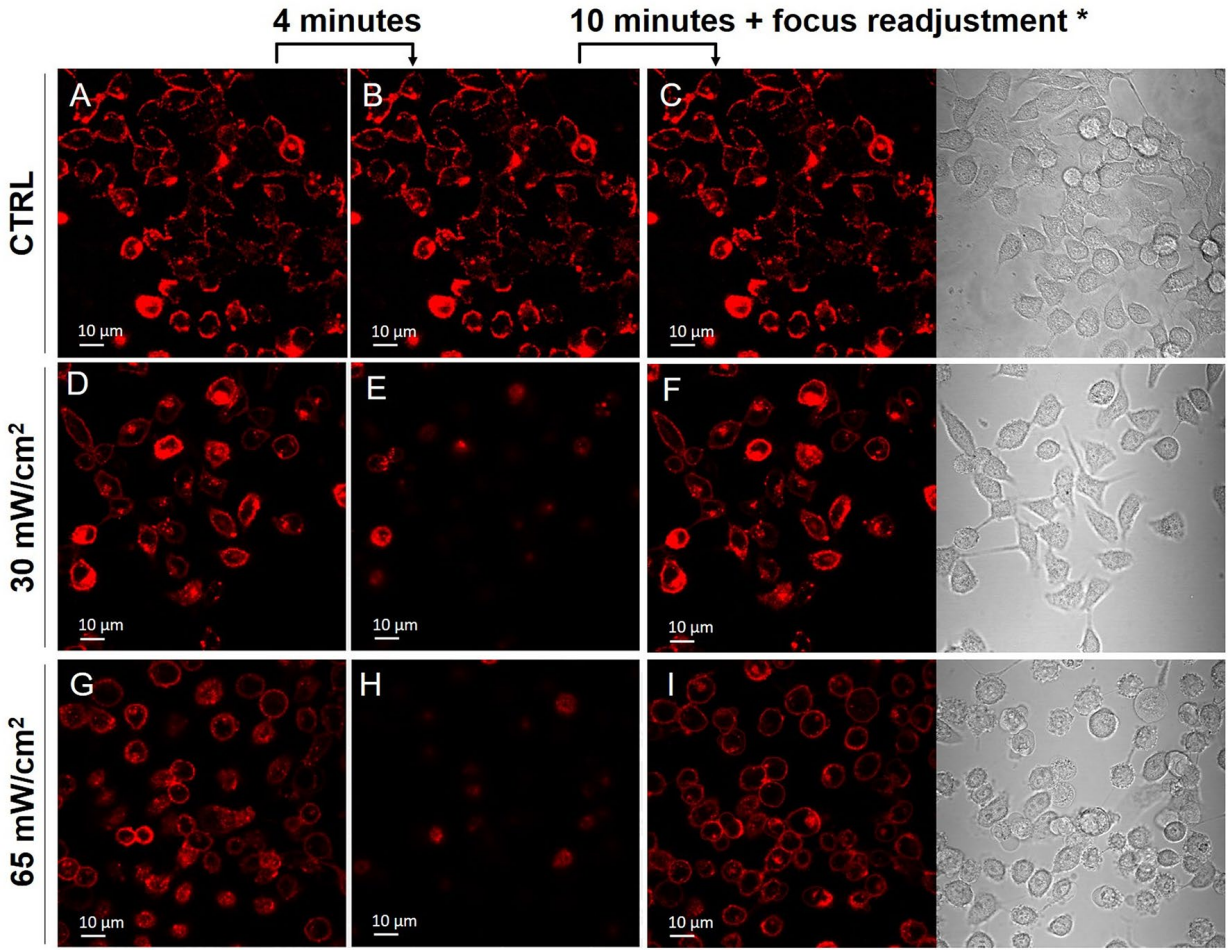
CLSM analysis of the morphology and positioning of HaCaT cells treated for 1 h with 1 MHz US using different acoustic intensities. For each I_spta_ value, we acquired at 10 min time-lapse immediately after the US treatment. The first frame of each acquisition is reported in the first column (**A**,**D**,**G**), and the corresponding frames acquired in the same region after 4 min of time-lapse, in the same focus plane of the first image, are reported in the second column (**B**,**E**,**H**). The frames reported in the last column were acquired in the same regions of the previous columns, at the end of the time-lapse and after readjusting the focus of ~ 13 μm towards the bottom of the Petri dish to match the new position of cells (**C**,**F**,**I**); the corresponding transmission microscopy images are also reported in the last column. Each time-lapse acquisition is representative of at least three different experiments. *For the control sample, no focus readjustment was needed.

Our study on cells treated with 1 MHz US is reported in Section S4 of ESI. The most relevant results, obtained in the same exposure conditions (1 h treatments at I_spta_ = 30 mW/cm^2^ and I_spta_ = 65 mW/cm^2^) that induce significant alteration of the membrane permeability and IL-6 up-regulation, are highlighted in Fig. 8. The time-lapse images show a progressive disappearance of cells during the first few minutes (Figure S5, cells disappear after 2–4 min). This effect is not observed in the control sample while it is slower (occurring after 6–10 min) and less pronounced in cells treated with I_spta_ = 18 mW/cm^2^ (Figure S4). Since all frames are acquired at fixed focus plane, these progressive changes could reflect precisely vertical movements of cells toward their stationary conditions, while remaining in adhesion to the Petri dish substrate. To confirm this hypothesis, at the end of each 10 min time-lapse, we moved the focus plane of the microscope towards the substrate to recover the image of the cells. Focus readjustment was not necessary for the control sample (panel C). The images acquired in the new position (Δz ≅ 13 μm), both by CLSM and transmission microscopy, confirm that cells are still in adhesion (panels F and I, see also Figure S4 for 18 mW/cm^2^ treatments), coherently to what observed in Fig. 7. This also rules out alternative possible explanations of the observed disappearing of cells, such as progressive detachment or probe artifacts (e.g., loss of DiI molecules and bleaching).

For cells treated at I_spta_ = 65 mW/cm^2^, the vertical movements are accompanied by evident morphological alterations, with membranes appearing significantly more round-shaped as compared to the control (compare panels A and panel G of Fig. 8) and to the image of the same sample acquired immediately before US exposure (panel D of Figure S5). However, after focus readjustment cells appear still in interaction with each other and with the substrate, confirming that the vanishing from the focus plane observed during time-lapses deals with cells that are in adhesion. This phenomenon may be plausibly explained by a progressive morphology “recovery” (e.g., turning back to their own more flattened shape).

For the sake of completeness, we have also documented qualitatively the viability of the DiI-labeled cells by calcein acetoxymethyl (AM), a non-fluorescent molecule, permeable to the cell membrane. When its acetoxymethyl ester group is hydrolysed by intracellular esterases, calcein AM acquires fluorescence, with excitation wavelength at 495 nm and emission wavelength at 515 nm (visible in green) and remains trapped in the cytosol if the plasma membrane is intact. This is a sign of active cellular metabolism. Our microscopy study on cells exposed to US at the different exposure conditions used in this work is reported in Figure S6. Images show that after the recovery time, most of the cells are still in adhesion (according to Figures S4, S5) with intact plasma membrane and are viable. However, we detected some dead cells that are still in adhesion, rarely after the exposures at I_spta_ = 30 mW/cm^2^ (panel E), and more often at I_spta_ = 65 mW/cm^2^ (panels G,H). Corresponding images at higher magnification are also shown in Figure S7. This is in qualitative agreement with the assays of viability and cell cycle reported above. In this line, Annexin V/PI and BAX/BCL-2 analyses suggest that these non-viable cells would undergo apoptosis.

## Discussion

The extensive clinical application of US in both diagnosis and therapy suggests the need of better understanding the related biological effects. Of particular interest is the phenomenon of transient cell poration induced by low intensity megasonic fields, a repairable alteration of the plasma membrane permeability induced by mechanical stress^1,11^. This effect is exploited in novel non-invasive drug and gene delivery strategies^2,3^.

Skin represents the first tissue that is exposed to the US stimulus. Keratinocytes are the principal cellular constituents of skin, which directly interact with external biochemical and biophysical stimuli and produce many cytokines capable of regulating the immune responses. In this respect, it is interesting to further investigate the bioeffects of transient membrane sonoporation on keratinocytes. To this aim, the present work is focused on 1 MHz LIPUS which are widely used in biological research and in therapeutic applications^52^ to convey mechanical energy through the skin tissue. Alongside sonoporation and cell viability effects, additional, yet undisclosed, IL-6 modulation on human keratinocytes could be activated. Specifically, our in vitro setup provides reproducible acoustic waves at the frequency of 1 MHz, exposure times up to 60 min, and low I_spta_ values that are associated to peak negative pressures of about 0.1 MPa in pulsed regime (10% duty cycle and pulses of 300 μs). These exposure conditions fall within those studied in the literature reporting effects of LIPUS on the plasma membrane structure^6,8,11,38^, and on the cell biology^5,6^, which could be reconducted to hydromechanical mechanisms.

We studied the effects induced by LIPUS in such exposure conditions on HaCaT cells, spontaneously immortalised cells that exhibit many similarities to primary basal keratinocytes but at the same time assure a high degree of reproducibility.

An increased production and secretion of IL-6 in culture media were pointed out in our experiments. The increase in protein levels correlated with the increased amount of IL-6 mRNA, indicating an up-regulation of IL-6 gene transcription. In addition, after US exposures, NF-κB, a transcriptional factor of IL-6, was rapidly detected in its active form in the cell nucleus. This result suggests that the applied mechanical stress could promote the increase in IL-6 secretion through the NF-κB signal pathway. It is worth noting that NF-κB activation and IL-6 up-regulation, that we reported in our experiments, were also detected in other cells exposed to mechanical stress^16,53^ as well to UV^39^ irradiation.

Proceeding from these results, we investigated the permeabilisation of the cell membrane induced by LIPUS exposure. In fact, it is extensively reported that non-thermal bioeffects may be elicited in cells exposed to acoustic fields in similar conditions. On the other hand, it is known that LIPUS exerts mechanical pressure on matter^6^, probably in ways similar to direct mechanical loading^54^ and fluidic shear stress^53–57^. Under such stimuli, the cellular constituents that operate as mechanosensors^58^ acting as linkers between the extracellular matrix molecules and the actin skeleton (e.g., integrins or stretch-activated ion channels) are activated^58–60^. Our sonoporation experiments pointed out the uptake of calcein, a fluorescent probe for the membrane integrity. The uptake efficiency increased both with the intensity of the applied US and with the exposure time. According to literature^8,38^ the higher the mechanical energy transferred to the cell membrane the deeper the sonoporation effect, facilitating the internalisation of large molecules within cells. In other words, the greater the size of the "transient pores" formed upon exposures the greater the diffusivity of the fluoroprobe into cells through the cell membrane. We would also stress that the observed increase in uptake efficiency with the exposure time is consistent with the sonoporation hypothesis, since the longer the exposure time the higher the probability of finding pores in the cell membrane that are large enough to allow access for calcein^8^. Moreover, according to literature, the membrane sonoporation could be related to some significant transient alteration of the cell morphology herein documented^51^. We suppose it might also involve cytoskeleton-membrane linkage^61,62^, leading to enhanced IL-6 production. On this line, it could be argued that 1 h exposure at 65 mW/cm^2^ might induce a transient alteration in the integrin-actin organisation, similarly to the effects reported by other studies^59–62^. Such alteration could consequently determine transient deformations and structural perturbations of the cell membrane (*e.g.*, reversible membrane poration), activating several signal pathways including NF-κB. On the other hand, at lower treatment doses (I_spta_ = 18 mW/cm^2^ and I_spta_ = 30 mW/cm^2^), the documented alterations in the morphology and permeability of the cell membrane are not accompanied by the up-regulation of the IL-6 secretion. This suggests that the LIPUS-induced bioeffects at the level of the cell membrane and on the cytoskeleton-membrane linkage have two different activation thresholds. An in-depth study in this respect, which is beyond the purpose of this work, would thus benefit from a direct evaluation of the cytoskeletal actins.

The concomitant increase in the BAX/BCL-2 ratio and in the Sub-G1 peaks, observed after 1 h exposure at 65 mW/cm^2^, points out that a small number of cells undergo apoptosis, as confirmed by Annexin V/PI and MTT assays. Together with such a decrease in the cell viability, we observed that US stress induces a transient deregulation of STAT3 signalling pathway, with a higher increase in unphosphorylated (U) STAT3 compared to pSTAT3, especially after 15 min of recovery time. A possible explanation would contemplate the hypothesis that STAT3 deregulation is related to the proliferative decrease revealed immediately after the LIPUS treatment. In addition, the accumulation of pSTAT3 detected after 3 h of recovery would instead reflect a resumption of cell proliferation. Undoubtedly, to better understand these results and to determine the fate of the involved HaCaT cells, a dedicated study should be carried out on the pathways activated during and immediately after exposure to US. However, our results emphasise the urgency of better understanding the role of US in the alteration of cellular homeostasis^63^.

Finally, mechanical stress induced by US resulted in a transient induction of IL-6 levels, but we did not observe the same effect on IL-1α, as highlighted by other studies^53^. Notice that the non-increase of extracellular IL-1α in the treated samples confirms that the evident phenomena of transient alteration of membrane permeability and cell morphology, observed at the highest LIPUS irradiation dose investigated here (1 h at I_spta_ = 65 mW/cm^2^), are not due to the formation of severe and irreversible damage to the structure of the cell membrane and necrosis. In fact, it is known that these events involve a significant extracellular release of IL-1α^64^.

Regarding the decrease in IL-1α we can refer to various explanations already reported in the literature. Indeed, it is known that this cytokine is chromatin-associated and translocates to the nucleus of living cells. Unlike, during apoptosis it was observed a reduced mobility of IL-1α for its concentration in dense nuclear foci^64^. As shown in Figure S1, we observed a biphasic response of IL-1α levels in HaCaT cells. IL-1α decreased after 3 h of post-treatment recovery, followed by a not statistically significant increase after 24 h. These results may suppose that the increase in apoptotic cells detected after US exposure is associated with the lacking release of IL-1α in treated HaCaT cells. In addition, these findings highlight that the IL-6 expression and secretion is not mediated by IL-1α, but probably by NF-κB activation. Some authors also observed^55^ that mechanical stress induced an increase of only IL-6 but not IL-1α, probably due to an increase in IL-1α receptor antagonist^65^.

As stressed in previous works^20^, our results focus attention on the need to reconsider biological damage indicators, to find others more representative of the effects induced by LIPUS at the cellular level. In light of the present results, it could be hypothesised that the modulation range of 1 MHz LIPUS exposure parameters could intersect the threshold doses of mechanical stress beyond which transient differential bioeffects could be triggered on keratinocyte models. The progressive and relevant activation of these bioeffects with increasing exposure doses in HaCaT cells would pass through slight morphological and membrane permeability changes, alterations of cell proliferation and the appearance of significant increase of IL-6 probably induced by activation of NF-κB. In this respect, it would be important to dedicate efforts in studying the biological response to exposures at different frequencies, which correspond to different mechanical indices.

## Conclusions

The present work identifies the stress conditions from ultrasonic exposures of biomedical relevance capable of activating proinflammatory pathways in vitro on a human keratinocyte model. The topic of this work was especially addressed to IL-6, highlighting a significant transient deregulation of IL-6 expression and secretion after 1 h exposure to 1 MHz LIPUS at I_spta_ = 65mW/cm^2^. Under the same conditions some increase of apoptosis and transient sonoporation phenomena have been identified. These results can pave the way to further investigating the molecular mechanisms induced by LIPUS in keratinocytes as well as in other cell types, and for longer exposure times.

In fact, it is known that a complex cytokine network drives proliferation and differentiation in keratinocytes^21^ and the deregulation of such cytokines signalling pathway is correlated to several chronic inflammatory skin diseases such as psoriasis, atopic dermatitis, and allergic contact dermatitis^66,67^. Thus, cytokines represent a potential target for therapy in skin inflammation^68^. In addition, IL-6 secretion induced by US can be released into the intercellular space and influence the maintenance of immunological response and disease progression correlated to local inflammation.

## Materials and methods

### Cell lines and culture conditions

The immortalised human keratinocyte cell line HaCaT, obtained from Interlab Cell Line Collection (Istituto Nazionale per la Ricerca sul Cancro, Genoa, Italy), was cultured in Dulbecco’s Modified Eagle Medium (DMEM) (Corning, Manassas, VA, USA) supplemented with 1% penicillin/streptomycin, 10% fetal bovine serum (FBS) and 1% l-glutamine (Euroclone, Pero, Italy). Cells were maintained in a tissue culture incubator at 37 °C, 5% CO_2_.

Cells were seeded in 9.6 cm^2^ cell culture-treated Petri dishes (Falcon^®^ Easy GripTM, Becton Dickinson Labware, Franklin Lakes, NJ; © ibidi GmbH, Martinsried, Germany) for 24 h at a density of 3–4 × 10^5^ cells/dish.

### Ultrasound exposure system and experimental protocol

The homemade setup employed for US exposures was previously described^38^^.^ This setup is capable of providing well defined sinusoidal waves independently from the exposure conditions. Briefly, it consists of a signal generator (Agilent 33220 A), a signal amplifier (Amplifier Research 25A250) and a submersible piezo-ceramic circular transducer (Precision Acoustics Ltd) with diameter of 44 mm tuned at 1 MHz.

The transducer is placed at the bottom of a tank (30 × 30 × 30 cm) filled with degassed Milli-Q water (18.2 MΩ·cm, resistivity). A cell culture-treated polystyrene Petri dish with diameter of 35 mm, containing cells in 2 mL of cultured media, is positioned at the water surface, submerged up to half of its thickness. The Petri dish is hermetically sealed and vertically aligned with the transducer. The thicknesses of the Petri bottom were ~ 0.90 mm (Falcon) or ~ 0.17 mm (ibidi), the latter allowing the use of high-resolution immersion objectives for CLSM. Acoustic absorbers were employed to suppress waves reflected from the tank and the temperature of the water bath was kept constant at 25 °C. The geometry of the Petri dishes and the overall exposure setup pattern were accurately chosen to avoid resonance effects with the wavelength of the US wave. These exposure conditions have been chosen to exclude or make ineffective any perturbation of the US field that can affect the reproducibility of experiments or generate in vitro response artefacts^69^.

Sample irradiation was operated in pulsed mode with 10% duty cycle and pulses of 300 μs keeping the Source-dish Surface Distance (SSD), i.e., the distance between the Petri dish and the transducer, constant at 7 cm to minimise close-field effects.

The acoustic intensity was calibrated by measuring the field transmitted from the Petri dish bottom area (where the cells are located) at different exposure conditions with a needle hydrophone of 0.5 mm diameter (Precision Acoustics, UK). During the calibration the setup configuration was exactly the same used for cell irradiation. The hydrophone was positioned inside the Petri dish using a micrometric slide, carefully placing it in close proximity to the bottom to accurately reproduce the experimental conditions and to account for any effect on the acoustic intensity due to the specific geometry of the exposure setup^38^. The same procedure of calibration was applied with the Falcon^®^ chambered cell culture slides, used in the immunofluorescence experiments. Intensities are provided in terms of I_spta_, i.e. the maximum spatial intensity measured when the pulse is activated, averaged for the period of pulse repetition. In all the experiments we used I_spta_ values ranging between 18 and 65 mW/cm^2^, below the cavitation threshold of 100 mW/cm^2^, and exposure times at 30 min and 60 min. Variability of the I_spta_ was within 10% thus providing a stable and reproducible US field calibration.

For each US treatment several control samples were analysed, placing them in the same environmental conditions of samples for an amount of time corresponding to US exposure time.

### Confocal laser scanning microscopy

Confocal laser scanning microscopy (CLSM) images were acquired using a Nikon Eclipse (Ti-E C1) inverted confocal microscope (objectives 20 × , 40 × , 60 × and 100 ×) equipped with an Argon ion laser (Spectra Physics, Mountain View, California) emitting at 488 nm, a He–Ne laser (Melles Griot, Florence, Italy) emitting at 543 nm, and a motorised stage. The same instrument was used to acquire transmission microscopy images.

For time-lapse sequences, the microscope allows to fix the focus plane position along the axial (*z*) direction with resolution of about 500 nm and thus to acquire a time-progression of images with a 30-s time interval.

The US induced alteration of the cell membrane permeability was characterised using the fluorescent green dye calcein (Sigma-Aldrich Italy, excitation and emission wavelengths at 495 and 515 nm, respectively). Lipid bilayers are impermeable to calcein, hence the presence of the green dye into the cell points out membrane poration, which allows calcein internalisation. Before applying US, 2 mL of 10 µM calcein solution in phosphate buffer (PBS) was added in the Petri dish containing cells; 10 min for membrane recovery were scheduled after treatments, and only then samples were rinsed in PBS and analysed by CLSM. The uptake efficiency was evaluated by the percentage of the cells in the sample in which it is possible to individuate internalised calcein.

Effects on cell morphology and positioning were studied using the fluorescent dye DiI. For labelling the cell membrane, before US exposure 10 µL of 1 mM solution in dimethyl sulfoxide (DMSO) were diluted in PBS to a final DiI concentration of 5 µM, which was added in the Petri dish containing cells and incubated for 5 min at room temperature under gentle stirring; excess dye was then removed by several rinsing with PBS. Notice that cell staining remained unchanged for at least 1 h, as verified by control samples. The cell morphology of DiI-stained cells was monitored by fluorescence time-lapse before and after US exposure (see also Section 4 of ESI). Time-lapse micrographs were acquired immediately after irradiation with 30-s time intervals for 10 min.

The viability of DiI stained cells was studied by labelling with calcein AM. For cell staining, a 4 mM solution of calcein AM was diluted 1000 times in PBS (2 µL in 2 mL total) to a concentration of 4 µM and added to the cells exposed to US, after 10 min of recovery. Cells were then incubated in the dark for 30 min at 37 °C. At the end of the incubation, cells were washed extensively with PBS and observed by CLSM. Unless otherwise indicated the samples incubated with Calcein AM were co-stained with DiI to delimit the cell membrane and better visualise the presence of non-viable cells within the culture.

Each CLSM experiment reported in this work was repeated on at least three different cell samples. Except for time-lapse studies, at least five different areas of each sample were analysed.

### Real-time qPCR

Total mRNA was isolated using a Total RNA Purification Kit (Norgen Biotek, ON, Canada) according to the manufacturer’s instructions. 1 µg of total RNA was converted into cDNA with M-MULV Reverse Transcriptase (New England BioLabs, UK). Real-time PCR was performed by Rotor-Gene Q (Qiagen, Hilden, Germany) using the BrightGreen qPCR MasterMix (abm, BC, CANADA). Specific forward and reverse primers were employed, having the following sequences: GAPDH F: 5′-GAAGGTGAAGGTCGGAGTC-3′, R: 5′-TTGAATGGCAACAATATCCACTT-3′; IL-6 F: 5′-AAGCCAGAGCTGTGCAGATG-3′, R: 5′-GTCCTGCAGCCACTGGTTCT-3′; BAX F: 5′-CAAGAAGCTGAGCGAGTGTCT-3′, R: 5′-GGTTCTGATCAGTTCCGGCAC-3′; BCL-2 F: 5′-TACGATAACCGGGAGATAGTGA-3′, R: 5′-CAGGTGCCGGTTCAGGTACT-3′. The expression of each gene was normalised to the expression of the glyceraldehyde-3-phosphate dehydrogenase (GAPDH) as reference gene and the relative mRNA concentrations were calculated using the ∆∆Ct method.

### Analysis of IL-6 and IL-1ɑ release by ELISA

For the analysis of ILs secretion in HaCaT cells, an amount of 3 × 10^5^ cells/dish was seeded into 9.6 cm^2^ tissue culture dishes. After cell attachment, cultured media was replaced, and the keratinocytes were exposed to the US for 1 h. After the US treatments, cells were maintained in the incubator at 37 °C, 5% CO_2_ for a recovery time of 15 min, 3 h, or 24 h. Control samples were maintained at the same environmental conditions of treated cells for an amount of time corresponding to treatment exposure time**.** Then, 1 mL of supernatant was harvested and centrifuged at 1200 rpm for 10 min to remove cells. Standards and replicates were prepared and assayed in triplicates for each sample. The release of IL-6 and IL-1ɑ in the supernatant was quantified by using an IL-6 High Sensitivity Human ELISA kit (Abcam, Cambridge, UK) and Human IL-1 alpha ELISA Kit (Sigma Aldrich, USA), respectively. Absorbance was measured at 450 nm with an ELISA plate reader (Labsystem Multiskan MS) and data were analysed using a standard curve generated from GraphPad prism (GraphPad software Inc, La Jolla, CA, USA). The levels of IL-6 and IL-1ɑ released were expressed as percent of the relative IL-6 and IL-1ɑ secreted in supernatant, comparing each sample to the corresponding control.

### Detection of NF-κB induction by immunofluorescence

HaCaT cells were seeded on Falcon^®^ chambered cell culture slides and, 24 h after cell attachment, exposed for 1 h to 1 MHz US with I_spta_ = 65 mW/cm^2^. After treatment, cells were washed with PBS and fixed with cold 4% buffered paraformaldehyde (Sigma-Aldrich) for 20 min at 4 °C, washed once with PBS and then permeabilised with 0.01% Triton X-100 for 5 min at room temperature. Cells were incubated for 1 h with 3% bovine serum albumin (BSA) (Sigma-Aldrich) at RT and successively with anti-NF-κB p65 mouse monoclonal antibody (Santa Cruz Biotechnology, CA, USA) diluted 1:25 for 1 h at RT^70^. Cells were then rinsed three times with PBS and incubated with Alexa Fluor 594 conjugated goat anti-mouse IgG (Jackson ImmunoResearch) diluted 1:200 for 1 h at RT. After rinsing with PBS, nuclear staining was achieved by 5 min of staining with Hoechst 33342 (10 μg/mL, Sigma-Aldrich) in PBS. Cells were finally rinsed twice in PBS, mounted with Prolong anti-fade reagent (Molecular Probes) and analysed using an Olympus BX52 (Hamburg, Germany) fluorescence microscope. The images were acquired and elaborated with IAS 2000 software (Delta Sistemi, Rome, Italy). Fluorescence intensity was analysed using Image J software (NIH Image): the densitometry of 10 randomly selected fixed squares from cytoplasm and nucleus of single cells was measured; the values were obtained in densitometric arbitrary units showing the mean optical density of each square and the mean values were calculated.

### Analysis of STAT3 and pSTAT3 by ELISA test

3 × 10^5^ cells were seeded in a Petri dish and after attachment culture media was removed. HaCaT cells were then irradiated for 1 h. After treatment, cells were harvested and lysed following the manufacturer's instructions. Standards and replicates were prepared and assayed in duplicates for each sample. The protein level of pSTAT3 and total STAT3 were detected by STAT3 (pY705 + Total) SimpleStep ELISA Kit (Abcam). Absorbance was measured at 450 nm with multimode microplate reader (Tecan Spark) and data were analysed using a standard curve generated from GraphPad Prism (GraphPad software). The levels of pSTAT3 and total STAT3 were expressed as pSTAT3/STAT3 ratio comparing each sample to the corresponding control.

### Cell proliferation analysis by MTT assay

5 × 10^3^ cells were seeded in 96-well flat plate and irradiated for 1 h. Cells were then incubated in FBS-free media with MTT for 4 h. Formazan crystals were then dissolved in 100 µL of DMSO and after 15 min the absorbance was measured at 540 nm in a plate reader spectrophotometer (Labsystem Multiskan MS). The absorbance of wells containing medium alone was subtracted to all samples.

Non-irradiated cells were used as control. Surviving cells were expressed as a percentage of the corresponding control.

### Cell cycle and apoptosis analysis by flow cytometry

Flow cytometry was used to analyse cell cycle and apoptosis by DNA staining and quantification using Propidium Iodide (PI). 5 × 10^5^ cells were seeded into 9.6 cm^2^ tissue culture dishes. After exposure to US, both adherent and floating cells were collected with an ice-cold PBS, fixed in cold 70% EtOH and incubated for 30 min at 4 °C. Then, the cellular pellet was washed twice in a phosphate-citrate buffer and treated with 50 µL of RNAse (100 µg/mL, Sigma) to ensure that only DNA was stained, and 200 µL of PI (50 µg/mL, Sigma) were added. Fixation of cells with precipitating fixatives causes the leakage of the cleaved low MW DNA fragments that are produced during apoptosis. As a consequence, apoptotic cells can be identified as a hypodiploid peak (Sub-G1), while healthy cells generate a typical cell cycle histogram^71^. Cell cycle analysis and Sub-G1 detection by quantification of DNA were performed by FACS CantoII (BD Biosciences). PI was excited by a 488 nm laser and emission of fluorescence was collected above 580 nm. Forward and right-angle scatter were used to define the cellular population and pulse processing of the PI signal was used to distinguish true G2 cells from G1 doublets. 10,000 single events were acquired for each sample.

In an independent experiment, controls and treated cells were collected, washed twice with PBS and resuspended in 1 × Annexin V Binding Buffer at 1 × 10^6^ cells/mL. 5 μL of Annexin V-FITC and 5 μL of PI were then added to cell suspension and incubated at room temperature for 15 min in the dark. After the incubation period, the stained cells were analysed by flow cytometry as previously described.

### Statistical analysis

All experiments reported in this work were performed in triplicate and data are expressed as mean value ± standard deviation. For CLSM at least five different areas of each sample were analysed. The results were analysed by one-way analysis of variance (ANOVA) and t test methods using GraphPad Prism 7.0 software. p < 0.05 values were considered statistically significant.

## Supplementary Information

Supplementary Information 1.
