# Supplementary material for: Effect of 1-MHz ultrasound on the proinflammatory interleukin-6 secretion in human keratinocytes
Source: Sci Rep. 2021 Sep 24;11:19033. doi: 10.1038/s41598-021-98141-2 (PMC8463532; doi:10.1038/s41598-021-98141-2)
Supplement: Supplementary file 1 — Supplementary Information 1. [file 41598_2021_98141_MOESM1_ESM.pdf]

Electronic Supplementary Information

Effect of 1-MHz ultrasound on the proinflammatory interleukin 6 secretion in human keratinocytes

Sabrina Giantulli,[1,†] Elisabetta Tortorella,[1,2,†] Francesco Brasili,[2,3,4] Susanna Scarpa,[5] Barbara Cerroni,[2] Gaio Paradossi,[2] Angelico Bedini,[6] Stefania Morrone,[5] Ida Silvestri[1,‡] and Fabio Domenici[2,‡,*]

[1] Department of Molecular Medicine, Sapienza University of Rome, Rome, Italy

[2] Department of Chemical Sciences and Technologies, University of Rome Tor Vergata, Rome, Italy

[3] Department of Physics, Sapienza University of Rome, Rome, Italy

[4] CNR-NANOTEC, Institute of Nanotechnology, Soft and Living Matter Laboratory, Rome, Italy

[5] Department of Experimental Medicine, Sapienza University of Rome, Rome, Italy

[6] Dipartimento Innovazioni Tecnologiche e Sicurezza degli Impianti, Prodotti e Insediamenti Antropici (DIT), INAIL, Monteporzio Catone, Rome, Italy

* Corresponding author: fabio.domenici@uniroma2.it

† Equally contributing authors

‡ Equally senior authors

S1. Effects of ultrasound on the secretion of IL-1α

To analyse the effects of ultrasound on the extracellular secretion of IL-1α, the ELISA test was performed at different recovery times on HaCaT cells exposed for 1 hour to 1 MHz ultrasound with I_{spta} = 65 mW/cm^2. The obtained results are reported in Figure S1. No statistically significant variations with respect to control samples were detected in treated cells, except for the sample analysed after a recovery time of 3 hours that shows a slight decrease.

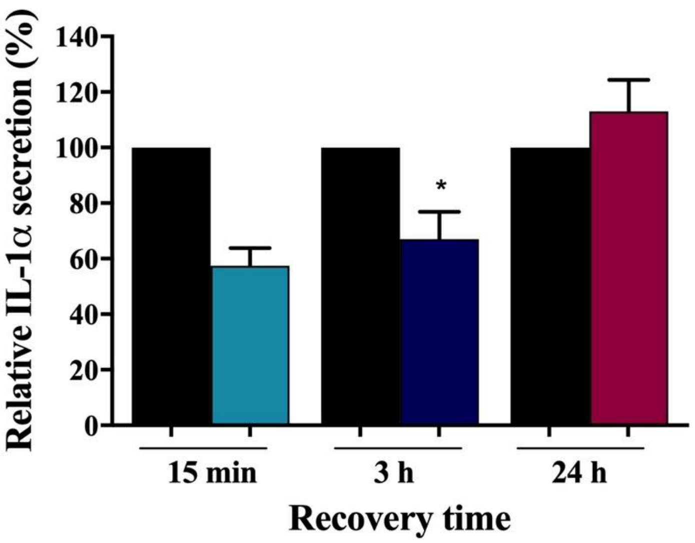

Figure S1. IL-1α secretion analysed by ELISA test performed on HaCaT cells exposed for 1 h to 1 MHz ultrasound at I_{spta} = 65 mW/cm^2, followed by different post-treatment recovery times. The control samples (black histograms) were maintained in the same environmental conditions of treated samples for an amount of time corresponding to exposure time. The reported values and error bars represent the average and the standard deviation evaluated on each sample analysed. Asterisks indicate significant difference from control samples (* $p<0.05$). The graph is representative of at least three independent experiments.

S2. Effects of ultrasound on the pSTAT3/STAT3 ratio

The expression of pSTAT3 and STAT3 proteins in HaCaT cells exposed for 60 minutes to 1 MHz ultrasound with I_{spta} = 65 mW/cm^2 was studied by the ELISA test. The cell lysate was analysed in comparison to the control sample using two different recovery times, 15 minutes and 3 hours, after treatments. Results are reported in Figure S2, revealing different pSTAT3/STAT3 ratios for the two recovery times. Referring to the control, a statistically significant decrease is observed after 15 minutes of recovery, while a slight increase occurs after 3 hours. These results are in accordance with flow cytometry, real-time PCR and MTT assay experiments, that highlighted a reduced cell proliferation. In addition, we detected an increase in the level of unphosphorylated (U)-STAT3 in treated cells. Several studies report that U-STAT3 can activate gene expression by different mechanisms with respect to pSTAT3 [1]. These results emphasize the importance of better understanding the role of the ultrasound in altering the cellular homeostasis and the STAT3 signalling pathway.

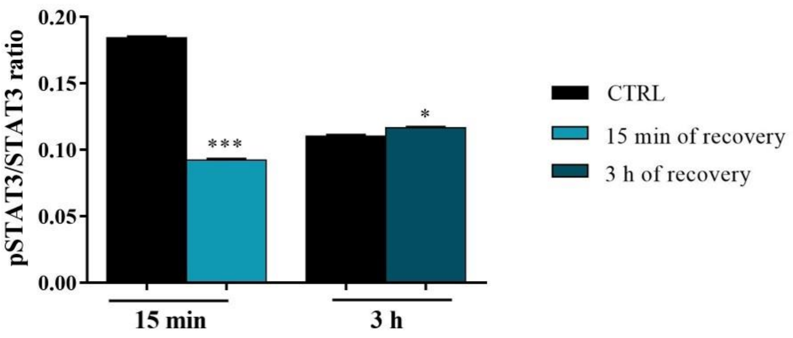

Figure S2. Analysis of the ratio between the protein expression levels of pSTAT3 and STAT3, measured by the ELISA test. HaCaT cells were exposed for 60 minutes to 1 MHz ultrasound with I_{spta} = 65 mW/cm^2. The cell lysate was then analysed after 15 minutes and 3 hours of recovery time. Each value and error bar represent the mean and the standard deviation calculated on three independent experiments; asterisks indicate significant difference from control samples (* $p<0.05$, *** $p<0.001$).

S3. Apoptosis analysis by flow cytometry

A flow cytometry analysis was performed using Annexin V/PI staining to evaluate the effects of ultrasound on the cell viability. The results obtained on HaCaT cells exposed for 60 minutes to 1 MHz ultrasound at varying the acoustic intensity are reported in panel A of Figure S3. The detail on the late-stage apoptosis, reported in panel B, highlights an increase of the revealed events with the applied intensity. This result is in accordance with the cell cycle analysis reported in the main text.

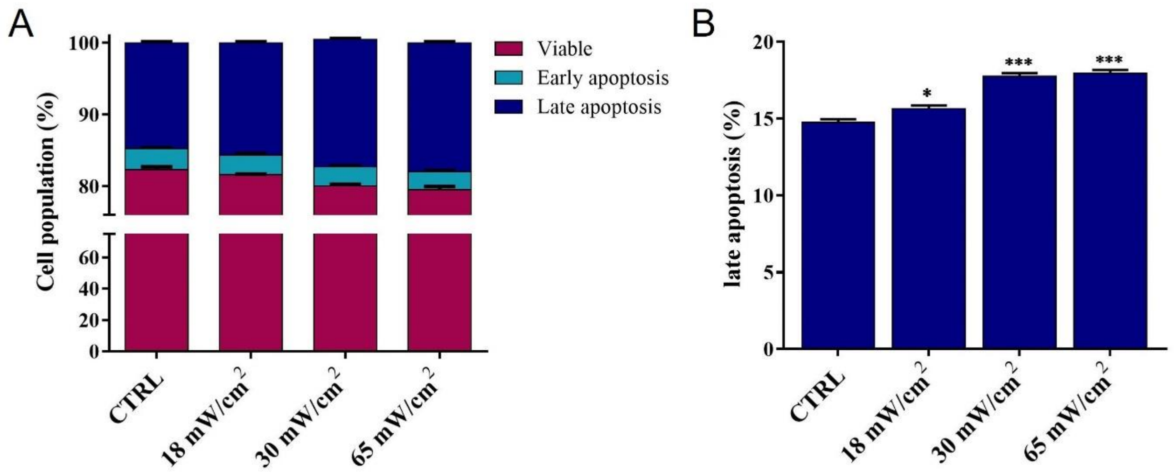

Figure S3. Annexin V/PI assay for analysing the apoptosis in HaCaT cells exposed for 60 minutes to 1 MHz ultrasound with different acoustic intensities. Each value and error bar represent the mean and the standard deviation calculated on three independent experiments; asterisks indicate significant difference from control samples (*$p<0.05$, *** $p<0.001$).

S4. Ultrasound induced variations in the shape and position of the cell membrane

Representative time-lapse images of samples underwent 1 MHz ultrasound with I_{spta} = 18 mW/cm^2 for 30 and 60 minutes, acquired by Confocal Laser Scanning Microscopy (CLSM) immediately after exposure, are reported in Figure S4 in comparison to control samples. An apparent progressive cell disappearance from the fixed focus plane is observed only in samples treated with ultrasound. This effect appears more marked in the sample exposed for 60 minutes (panel E), where most of the cells fall out of the fixed focus plane after 4 minutes from ultrasound switching off. The apparent shift in the vertical position of the cell membrane profile may reflect a progressive change in the overall cell shape and positioning. To check this hypothesis, after 10 minutes of time-lapse, we moved the focus plane of the microscope of a few microns towards the Petri dish substrate to recover the cells. We did not readjust the focus for control samples. The images acquired in the new position, both by CLSM and transmission microscopy, are reported in the figure (panels B, D and F). They show that cells are still in adhesion, therefore a shape "recovering" (*i.e.*, cell flattening on the substrate) might have occurred during the time-lapse acquisition.

Representative time-lapses acquired on samples irradiated using higher intensities (I_{spta} = 30 mW/cm^2 and I_{spta} = 65 mW/cm^2) for 60 minutes are reported in Figure S5. The same effect of vertical shift occurs also in this case, more rapidly for the highest intensity, at which most of the cells are no more visible already after 2 minutes. Notice that immediately after the treatment at I_{spta} = 65 mW/cm^2 (first image of panel E) the membrane shape of the HaCaT cells appears significantly more round-shaped with respect to the control acquired just before exposing the same sample to ultrasound (panel D), and even to samples treated at lower intensities (first image of the time-lapses reported in panels B and E of Figure S4). After 10 minutes of time-lapse, the cells treated using both 30 mW/cm^2 and 65 mW/cm^2 intensities resulted vertically shifted of ~13 μm towards the Petri substrate (panels C and F of Figure S5). In the case of 65 mW/cm^2 treatments, the same conditions that induce the alterations in cell viability and in IL-6 secretion reported in the main text, cells still appear round-shaped. However, cells appear in adhesion to Petri dish substrate. In support of this, we also performed in Section S5 a qualitative check of the viability of such DiI-stained cells using the calcein acetoxymethyl (AM) viability probe.

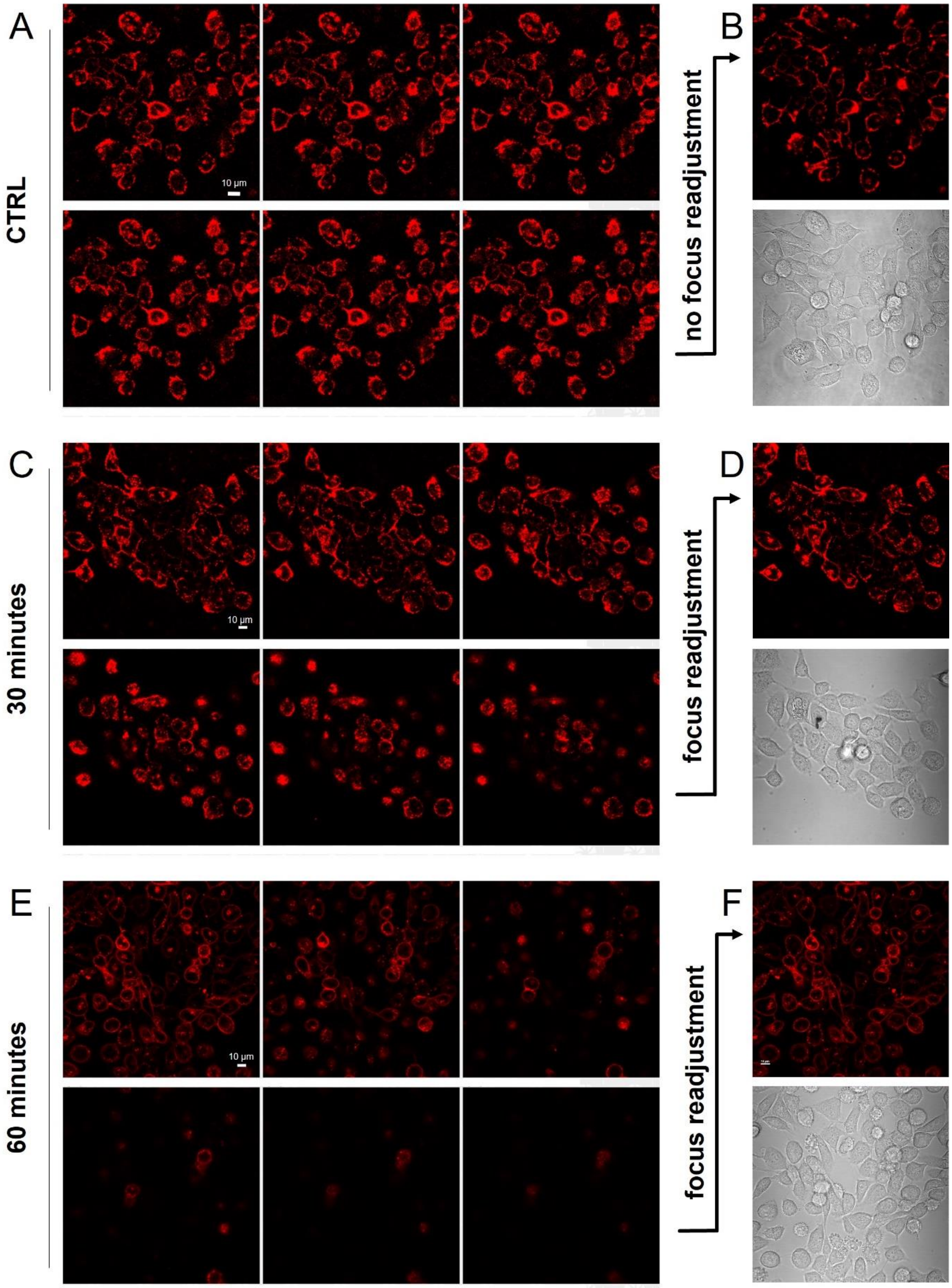

Figure S4. Time-lapse study of the position and membrane morphology of HaCaT cells treated with 1 MHz ultrasound with I_{spta} = 18 mW/cm^2 for 30 and 60 minutes. For each sample, in the first column (A, C, E) one frame every 2 minutes is shown, from top left (0 minutes) to bottom right (10 minutes). The frames reported in the second column (B, D, F) have been acquired in the same region of the previous column, at the end of the time-lapse and after readjusting the focus to match the new position of cells (except for the control sample, where no focus readjustment was needed); the corresponding transmission microscopy images are also reported.

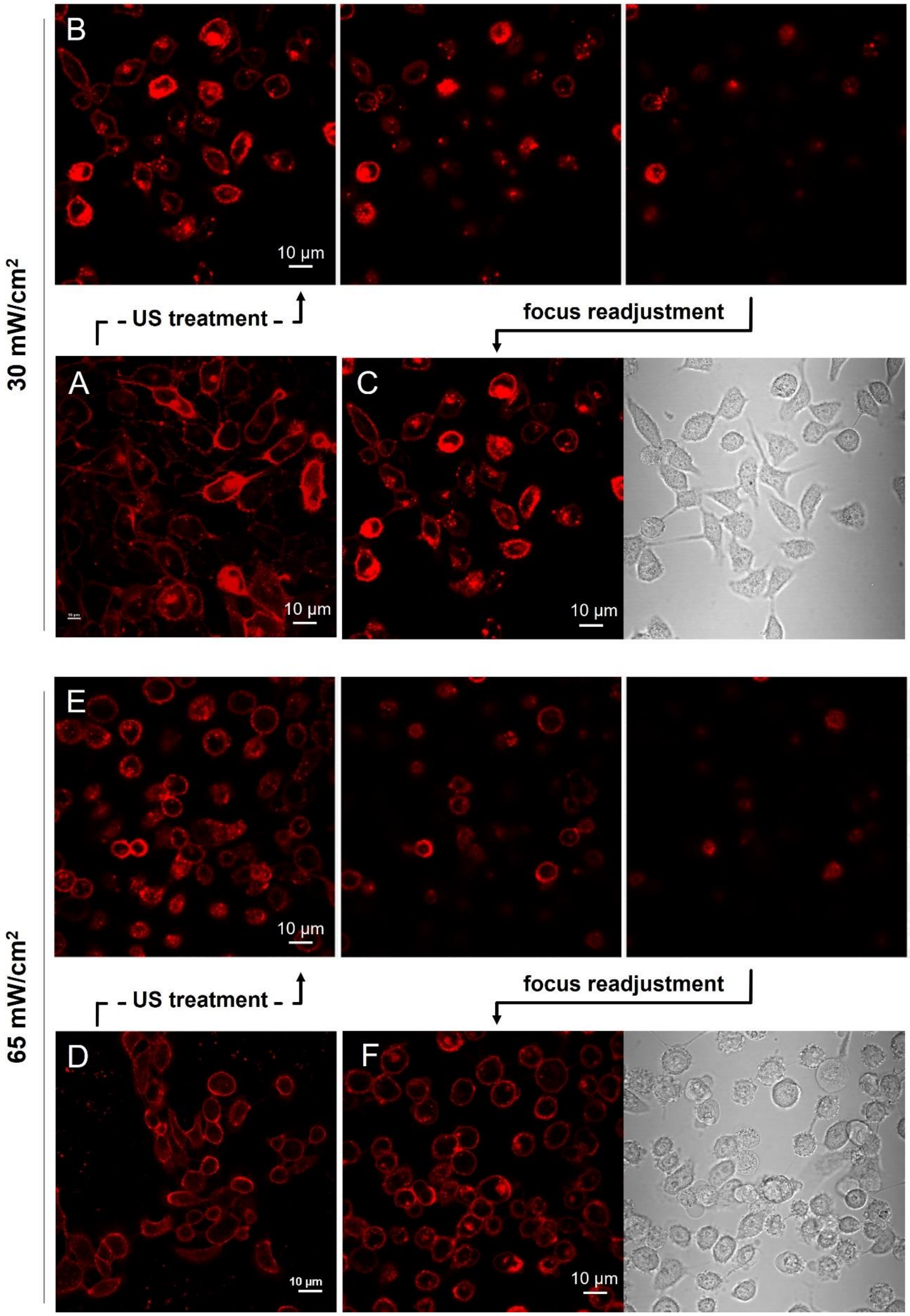

Figure S5. CLSM time-lapse study of the position and membrane morphology of HaCaT cells treated for 1 hour with 1 MHz ultrasound at I_{spta} = 30 mW/cm^2 and I_{spta} = 65 mW/cm^2. For each intensity, a representative image acquired just before exposure (A, D), the time-lapse acquired immediately after exposure (B, E), and the image acquired at the end of the time-lapse in the same region and after readjusting the focus of ~13 µm to match the new position of cells (C, F), together with the corresponding transmission microscopy image, are reported. For time-lapses, one frame every 2 minutes is shown.

S5. CLSM analysis of the HaCaT cells viability

The viability of HaCaT cells underwent ultrasound treatments was studied by staining with calcein AM. Calcein AM is a non-fluorescent dye, permeable to the cell membrane. When its acetoxymethyl-ester group is hydrolyzed by intracellular esterases, calcein AM acquires fluorescence, with excitation wavelength at 495 nm and emission wavelength at 515 nm (visible in green). This is a sign of active cellular metabolism. For cell staining, a 4 mM solution of calcein AM was diluted 1000 times in PBS (2 µl in 2 mL total) to a concentration of 4 µM and added to the cells exposed to ultrasound, after 10 minutes of recovery. Cells were then incubated in the dark for 30 minutes at 37 °C. At the end of the incubation, cells were washed extensively with PBS and observed by CLSM. We documented the cell viability for the different exposure conditions in Figure S6 and Figure S7. The green fluorescence is associated to viable cells with intact plasma membrane. Except for the control sample of Figure S6A (30 minutes, no exposure to ultrasound), all samples were co-stained with DiI to delimit the cell membrane and better visualize the presence of non-viable cells within the culture. According to the reported images, it can be confirmed that after the recovery time cells are viable and in adhesion. However, rarely after the exposures at I_{spta} = 30 mW/cm^2 (Figures S7A and S7B) and more often for I_{spta} = 65 mW/cm^2 (Figures S6H, S7C and S7D), some dead cells which are still in adhesion can be detected.

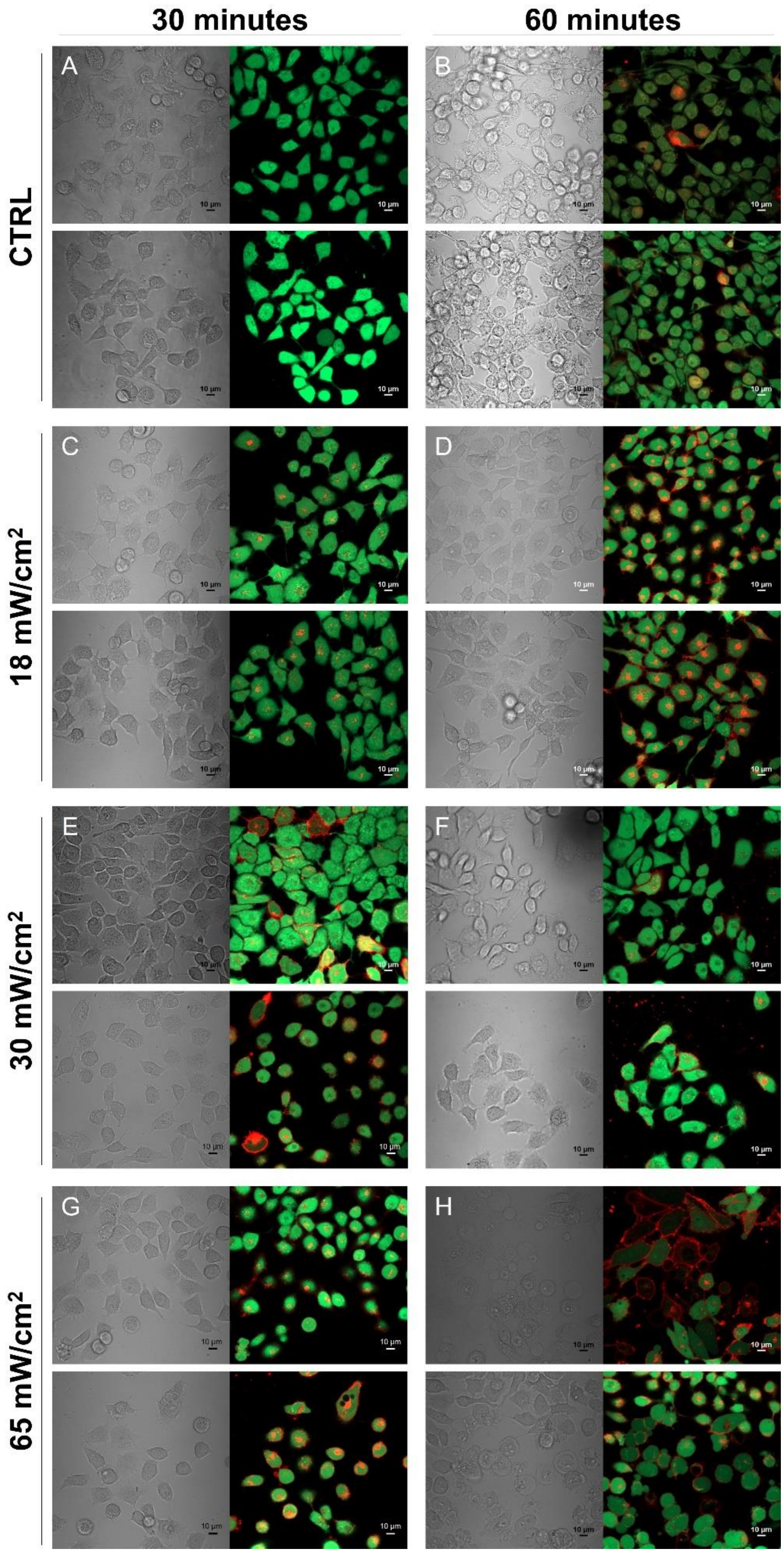

Figure S6. CLSM study of the viability of HaCaT cells exposed to 1 MHz ultrasound with different intensities and treatment times. Viable cells are identified by the green fluorescence, turned on as a consequence of the hydrolysation of calcein AM by intracellular esterases. Except for the control sample of 30 minutes treatments (panel A), the cell membranes can be visualised in red due to DiI staining.

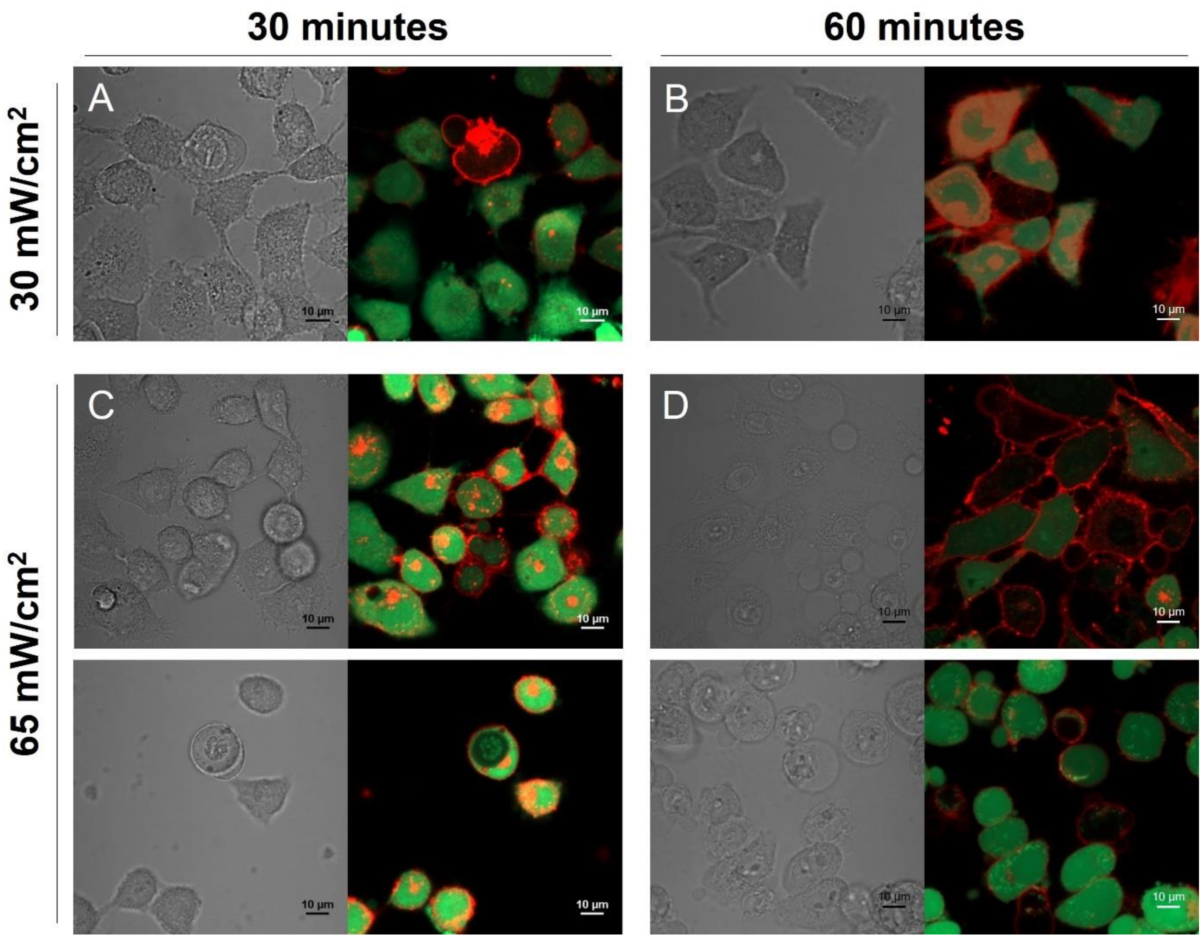

Figure S7. CLSM study of the viability of HaCaT cells exposed to 1 MHz ultrasound with different intensities and treatment times. Images were acquired at higher magnification with respect to Figure S6, to highlight some dead cells that can be rarely detected in samples treated with the highest acoustic doses. Viable cells are identified by the green fluorescence, turned on as a consequence of the hydrolysation of calcein AM by intracellular esterases. The cell membranes can be visualised in red due to DiI staining.

References

[1] Lizzul, P. F. *et al.* Differential expression of phosphorylated NF-κB/RelA in normal and psoriatic epidermis and downregulation of NF-κB in response to treatment with etanercept. *J. Invest. Dermatol.* 124, 1275-1283 (2005).
